# The Beyond Ageing Project Phase 2 - a double-blind, selective prevention, randomised, placebo-controlled trial of omega-3 fatty acids and sertraline in an older age cohort at risk for depression: study protocol for a randomized controlled trial

**DOI:** 10.1186/s13063-015-0762-6

**Published:** 2015-06-03

**Authors:** Nicole L. Cockayne, Shantel L. Duffy, Rosalind Bonomally, Amelia English, Paul G. Amminger, Andrew Mackinnon, Helen M. Christensen, Sharon L. Naismith, Ian B. Hickie

**Affiliations:** Healthy Brain Ageing Program, Brain and Mind Research Institute, University of Sydney, 94 Mallett Street, Camperdown, NSW 2050 Australia; Orygen - The National Centre of Excellence in Youth Mental Health, The University of Melbourne, Locked Bag 10, Parkville, VIC 3052 Australia; Black Dog Institute, University of New South Wales, Hospital Road, Prince of Wales Hospital, Randwick, NSW 2031 Australia

**Keywords:** Depression, Prevention, OMEGA-3 fatty acid, Antidepressants, Randomised controlled trial, Older adults

## Abstract

**Background:**

Late-life depression is associated with high rates of morbidity, premature mortality, disability, functional decline, caregiver burden and increased health care costs. While clinical and public health approaches are focused on prevention or early intervention strategies, the ideal method of intervention remains unclear. No study has set out to evaluate the role of neurobiological agents in preventing depressive symptoms in older populations at risk of depression.

**Methods/Design:**

Subjects with previously reported sub-threshold depressive symptoms, aged 60 to 74 years, will be screened to participate in a single-centre, double-blind, randomised controlled trial with three parallel groups involving omega-3 fatty acid supplementation or sertraline hydrochloride, compared with matching placebo. Subjects will be excluded if they have current depression or suicide ideation; are taking antidepressants or any supplement containing omega-3 fatty acid; or have a prior history of stroke or other serious cerebrovascular or cardiovascular disease, neurological disease, significant psychiatric disease (other than depression) or neurodegenerative disease. The trial will consist of a 12 month treatment phase with follow-up at three months and 12 months to assess outcome events. At three months, subjects will undergo structural neuroimaging to assess whether treatment effects on depressive symptoms correlate with brain changes. Additionally, proton spectroscopy techniques will be used to capture brain-imaging markers of the biological effects of the interventions. The trial will be conducted in urban New South Wales, Australia, and will recruit a community-based sample of 450 adults. Using intention-to-treat methods, the primary endpoint is an absence of clinically relevant depression scores at 12 months between the omega-3 fatty acid and sertraline interventions and the placebo condition.

**Discussion:**

The current health, social and economic costs of late-life depression make prevention imperative from a public health perspective. This innovative trial aims to address the long-neglected area of prevention of depression in older adults. The interventions are targeted to the pathophysiology of disease, and regardless of the effect size of treatment, the outcomes will offer major scientific advances regarding the neurobiological action of these agents. The main results are expected to be available in 2017.

**Trial Registration:**

Australian and New Zealand Clinical Trials Registry ACTRN12610000032055 (12 January 2010)

**Electronic supplementary material:**

The online version of this article (doi:10.1186/s13063-015-0762-6) contains supplementary material, which is available to authorized users.

## Background

Depression is one of the most prevalent psychiatric conditions of later life, with clinically relevant depressive syndromes affecting 9 to 18 % of the general elderly population [1, 2]. Late-life depression (LLD) describes a major depressive episode occurring after the age of 60 or 65 years. It is associated with significant morbidity and mortality, and contributes to cognitive decline [3–5] and disability [6]. Research suggests that even with optimal treatment, only 34 % of the LLD disease burden in the form of ‘years lived with disability’ could be averted [7].

Whilst many older adults fail to meet criteria for major depression [8], subsyndromal depressive symptoms are clinically significant and are associated with cognitive impairment [9] and an increased risk for the development of major depression [10]. Longitudinal community studies show that even in those with subsyndromal depression, persistence or worsening of depressive symptoms occurs over time [9]. Importantly, both clinical [11] and epidemiological [12] studies show that either subsyndromal or full-threshold depressive disorders are risk factors for dementia. Even one clinically significant depressive symptom can increase an individual’s risk of dementia by up to 20 % [13], and a lifetime episode of depression increases dementia risk threefold [14]. Importantly, epidemiological research has shown that a reduction in major depression (MD) prevalence could reduce the incidence of Alzheimer’s disease by 7.9 % worldwide [15], and all cause dementia by up to 10 % [16]. The current health, social and economic costs of depression are such that, from a public health perspective, depression prevention should be seen as imperative, and with the world’s rapidly ageing population, the development of effective prevention strategies is now critical.

### The need for depression prevention trials

While the need to conduct prevention trials is widely recognised [9, 10], the ideal method of intervention is unclear. This may partly reflect the multi-faceted nature of LLD where lifestyle, behavioural, neurotoxic (*e.g.* glucocorticoid), genetic (*e.g.* via homocysteine), vascular, glial and inflammatory mechanisms are important contributors to pathophysiology [17, 18] (see also below). Additionally, it appears that once the illness is established, neurobiological changes are unlikely to be reversible [17]. Only one known study focusing on ‘at risk’ community older people has been conducted and utilised a stepped-care approach (watchful waiting, CBT-based self-help intervention, problem-solving treatment and referral to a primary care physician for medication if required). This study demonstrated a halved incidence of depressive and anxiety disorders in older adults presenting with sub-threshold depression and anxiety (not meeting diagnostic criteria) over a period of 12 months [19], and these effects were sustained at two years [20]. Recently, in Phase 1 of the BA trial, we found positive effects of folate on cognition [21], reinforcing strategies aimed at targeting underlying pathophysiology.

### Mechanisms underpinning brain change in depression

The mechanisms underpinning the link between LLD and poor longitudinal outcomes are not yet fully delineated, but a combination of vascular, inflammatory, illness-specific and neurodegenerative changes may be operative [22]. In recent years, the role of oxidative stress and inflammatory mechanisms in depressive disorders has been the subject of a plethora of studies. Indeed, altered levels of antioxidants and their synthesising enzymes, high concentrations of oxidation by-products and increased pro-oxidant activity levels have been demonstrated in patients with LLD and have also been linked with cardiovascular disease [23–31]. Furthermore, increased inflammatory markers have been demonstrated in patients with depression and comorbid cognitive impairment, with chronic inflammation thought to play a central role in dementia pathophysiology [23, 24, 28–31].

Depressive illness appears to further contribute to brain change, particularly in the hippocampus, a key structure involved in memory and neurogenesis. The ‘neurotoxic’ effects of depression may occur via down-regulation of the hypothalamic-pituitary adrenocortical axis, neurotoxic effects of glucocorticoids, and reduced expression of neurotrophins [32]. Indeed, our prior work has shown direct links between reduced hippocampal size and impaired memory performance in older people with depression [33]. Patients with depression have been shown to have reduced levels of brain-derived neurotrophic factor (BDNF) [32], a key neurotrophin relevant for hippocampal neurogenesis. The presence of depressive illness also appears detrimental to the integrity of brain structures via prolonged hypothalamus-pituitary-adrenal (HPA) axis activation [13, 14]. Preclinical studies have demonstrated that prolonged glucocorticoid exposure resulting from HPA axis hyperactivation may lead to increased free radical production, reduced glucose transport, a reduction in brain-derived neurotrophic factor (for review see [34]) and suppressed neurogenesis. Together, these effects, resulting from prolonged stress and hypercortisolaemia, have been posited to account for the reduction in hippocampal volume commonly associated with depression occurring across the lifespan [35], and has been associated with memory decline in older adults [33, 36]. Thus, in order to reduce the incidence of LLD and the concomitant cognitive decline, we argue that prevention should focus on the known underlying neurobiology including reduction of vascular/inflammatory risk factors and targeting ‘neuroprotection’ via promotion of neurotrophic factors [17]. Of relevance to this phase of our prevention study, the National Heart Foundation of Australia now recommends omega-3 fatty acid consumption for the prevention of cardiovascular disease, and a plethora of studies suggest that antidepressants may offer neuroprotection, the promotion of neurotrophins and neurogenesis.

### Omega-3 for prevention of depression and cognitive decline

Clinical and epidemiological studies suggest that low levels of omega-3 fatty acids (specifically eicosapentaenoic acid (EPA; precursor to DHA), and docosahexaenoic acid (DHA)) may occur in a number of psychiatric conditions, including depression. While there have been some negative findings [37], four controlled intervention studies have shown that omega-3 fatty acids reduce depressive symptoms in adults with major depression [38–41]. The size of the effect attributable to omega-3 fatty acid is unresolved due to the variability in sample size (20 to 452), measures of depression used and treatment duration (28 to 182 days), as well as the ratios of EPA and DHA.

With regard to cognitive benefits, longitudinal research suggests that whole fish consumption is protective against cognitive decline and dementia [42]. A recent prospective study, the Singapore Longitudinal Aging Study, showed that daily omega-3 fatty acid supplements were associated with lower risk of cognitive decline in a cohort of Chinese adults aged over 55 years [43]. Positive effects on processing speed appear to be particularly pronounced. For example, over a three year time period, higher plasma omega-3 fatty acid proportions were associated with less decline in cognitive tasks mediated by processing speed [44]. Over a 35-day period, positive effects of EPA/DHA have been noted in both mood and reaction time [45].

Despite this body of work focusing on the effects of omega-3 fatty acid supplementation on depressive symptoms and cognition, to date there have been no randomised controlled trials investigating whether supplementation can *prevent* depressive symptoms in older adults. Similarly, although animal studies show that long-term omega-3 fatty acid depletion can produce cognitive deficits in normal animals (for review see [46]), there is still uncertainty regarding the benefit of omega-3 fatty acid supplementation on cognitive decline and dementia [47]. However, given 39 % of Australians aged over 50 years report taking an omega-3 fatty acid supplement [48], clinical evidence of a therapeutic benefit from omega-3 fatty acid supplementation in the *prevention* of cognitive decline warrants further investigation.

### Potential mechanism of effect of omega-3

Omega-3 fatty acids are an integral component of cell membranes, facilitating membrane fluidity and permeability. They are necessary for brain function and development, and may have neuroprotective properties. Cell signalling and signal transduction, including serotonin and dopamine pathways, have been shown to be influenced by omega-3 fatty acids [49]. Omega-3 fatty acids are essential for the maintenance of cell structure and have anti-inflammatory effects; mediating and modulating acute inflammatory responses [50], and decreasing pro-inflammatory cytokine production [51, 52].

Animal studies have shown that supplementation with omega-3 fatty acids reversed age-related impairments in long-term potentiation and depolarization-induced glutamate transmitter release (impairments thought to arise as a result of a decrease in membrane fluidity). DHA is decreased in the hippocampus of aged rats and can be restored through dietary manipulation [53]. In addition, omega-3 fatty acid has been demonstrated to have a direct antioxidant effect, with supplementation in ageing rats shown to enhance antioxidant activity [54]. Importantly, a novel clinical study using magnetic resonance spectroscopy (MRS) has demonstrated that in patients with first-episode psychosis, omega-3 fatty acid supplementation (2 g EPA) is associated with significant increases in glutathione (GSH), which is the brain’s major antioxidant in comparison to treatment as usual [55]. As GSH protects dopaminergic neurons from oxidative and excitatory damage [56], such an increase suggests neuroprotective effects.

From a clinical perspective, omega-3 fatty acid supplementation is generally well tolerated, is associated with few undesirable side effects [41], has limited interaction with other medications and has been shown to improve cardiovascular and immune health [41, 57], factors that are particularly pertinent in older people. Thus, omega-3 fatty acid supplementation could be a suitable and safe prevention treatment for depression and cognitive decline in older adults [46, 58, 59].

### Antidepressants for the prevention of depression and cognitive decline

Evaluation of the role of antidepressants as prevention agents for depression has seen positive findings in patients with recurrent major depression [60], post-stroke depression [61, 62] and diabetes [63]. However there are no known studies in older ‘at risk’ adults. Due to concerns about the unwanted effects of antidepressants, psychological interventions have been favoured to date [20, 19]. However, as it is likely that there are key bi-directional relationships between depression, cognitive changes and structural brain changes, the potential neuroprotective and anti-inflammatory effects of antidepressants, and sertraline in particular, suggest a role for such treatments not only in the amelioration of depressive and cognitive symptoms, but also in their prevention. Of added benefit and significance, a large body of evidence suggests that antidepressants in older people are associated with reduced suicide rates [64].

### Sertraline as an indicated prevention intervention for depression

Sertraline, a selective serotonin re-uptake inhibitor (SSRI) is a specific and potent inhibitor of serotonin re-uptake at the presynaptic terminal with a modest inhibitory effect on dopamine reuptake. It has been shown to be effective in the treatment of major depressive disorder in older adults, with a dose range of 50-200 mg daily (for review see [65]), appears to be superior to placebo and has similar efficacy to other antidepressants (from different classes) [65]. Additionally, a recent animal study showed that sertraline has a protective action against cognitive decline in an induced neurodegenerative disease model [66]. In this study, rats treated with 3-nitropropionic acid performed poorly on follow-up assessments of memory retention and spatial navigation, whereas those concurrently treated with sertraline showed a preservation of baseline function. Due to the lack of anti-cholinergic effects as well as few interactions with other medications, sertraline is more tolerable than alternate classes of antidepressant [65]. A number of reviews suggest that sertraline is the most suitable first choice of antidepressant in patients suffering major depression due to its balance of efficacy and acceptability [67].

### Potential neuroprotective effects of selective serotonin re-uptake inhibitors

A growing body of literature suggests that SSRIs may have both neuroprotective and anti-inflammatory actions in the brain. Animal studies suggest that SSRIs may promote the growth of new neuronal pathways or neurogenesis [68]. Sertraline appears to increase neuronal differentiation as well as promote neuronal maturation of human hippocampal progenitor cells [69]. These effects may be related to the action of SSRIs to cause up-regulation of BDNF in the hippocampus [70]. SSRIs may owe some of their effect to anti-inflammatory actions on microglia [71]. Indeed sertraline has been shown to prevent GSH (the brain’s major antioxidant) reduction in rats with induced neurodegeneration [66], which is of particular significance given recent work showing that this antioxidant is linked to cognitive decline in older adults at risk of dementia [72].

### Summary and rationale for the second Beyond Ageing Project randomised controlled trial

Taken together, there is urgent need to conduct relevant prevention or early intervention trials for depression and cognitive decline in older adults, and substantial evidence indicates that omega-3 fatty acid supplementation and sertraline treatment may have therapeutic potential in this regard. As we are testing two novel pharmacological prevention treatments, the comparator of choice will be a placebo condition.

As such, we aim to conduct a selective prevention trial for depressive symptoms in an established and well-characterised cohort of older ‘at risk’ adults, who initially participated in the first Beyond Ageing Project (BAP) [73]. The BAP Cohort is a community-based population of older adults initially aged 60 to 74 years when recruited in 2005 to 2006 from the Australian Electoral roll. At that time, participants were recruited from urban and rural sites in New South Wales and the Australian Capital Territory, Australia, by postal mail, using address and age-range information provided by the Australian Electoral Commission. Given that voting is compulsory in Australia, this strategy provided for representative population sampling. Initially, these people were selected as being at elevated risk of major depression as evidenced by scores >15 on the Kessler-10 (K-10) questionnaire. The K-10 questionnaire is a self-report global measure of psychological distress and comprises ten items concerning anxiety and depressive symptoms experienced during the most recent 4-week period [74]. The K-10 is designed for use in community samples, and is useful for detecting levels of distress that are associated with independent diagnostic measures (for example, DSM-IV) in older adults [74]. A K-10 score >15 (moderate psychological distress) is found in 25 % of the aged population in Australia [75] across all age groups, and correlates with a 9.1 % 12 month prevalence of affective disorder.

Our rationale for the choice of participants is supported by longitudinal community studies that have demonstrated that for older people with subsyndromal depression, persistence or worsening of symptoms occurs over time [9]. Additionally, because people with depressive symptoms are prevalent in the community, selective prevention approaches are feasible. Our approach to target those ‘at risk’ of depression makes efficient use of resources since cohorts of subjects with subsyndromal depression yield the highest attributable risk, the lowest numbers needed to treat and the shortest time frames for testing for beneficial effects.

Our prior randomised controlled trial (RCT) with this BAP cohort showed that mental health literacy was associated with a decrease in depressive symptoms and that folate supplementation was associated with lowered rates of cognitive decline [73]. Here, we extend our prior work, and propose to evaluate the role of agents that target inflammation/oxidative stress, vascular health and neuroprotection.

### Objectives

The study aims to determine whether the following statements are true:

1. Omega-3 fatty acid or sertraline is associated with reduced rates of depressive symptoms or incident depression over a 12 month period;
2. Omega-3 fatty acid or sertraline is associated with reduced rates of cognitive decline over a 12 month period;
3. Markers of neuronal, glial and inflammatory brain changes predict an individual’s capacity to benefit from the omega-3 fatty acid or sertraline interventions; and,
4. Omega-3 fatty acid or sertraline is associated with specific changes in brain metabolism in-vivo as measured by Magnetic Resonance Spectroscopy (MRS).

### Hypotheses

Our hypotheses are as follows:

1. In comparison to placebo, participants receiving omega-3 fatty acids or sertraline will demonstrate reduced rates of depressive symptoms and reduced occurrence of depressive disorder at 12 months;
2. In comparison to placebo, participants receiving omega-3 fatty acids or sertraline will have less cognitive decline over 12 months in the domains of processing speed and memory, respectively;
3. Evidence of brain compromise (volumetric loss and/or changes in neurometabolites) will be associated with reduced efficacy of omega-3 fatty acid supplementation or sertraline for preventing depressive symptoms or cognitive decline; and,
4. Participants receiving omega-3 fatty acid and sertraline supplementation will demonstrate changes in inflammatory/oxidative stress markers (GSH) and neuronal integrity (Nacetylaspartate, NAA) as measured *in vivo* by MRS.

## Methods/Design

### Trial design

The study is a single-centre, double-blind RCT with three parallel groups involving two novel pharmacological treatments and a placebo condition. There will be three principal occasions of measurement over the 12 month treatment period: baseline, three months, and 12 months with the primary endpoint an absence of clinically relevant depressive symptoms at 12 months. Participants in all three arms will have a brief clinical review and collect investigational products monthly for the first three months. They will be contacted via telephone at the following intervals (one, two, six, and ten weeks and then monthly to 12 months) for safety monitoring and adherence purposes. The trial has been registered on the Australian and New Zealand Clinical Trial Registry (ACTRN12610000032055). An additional file depicts the trial design graphically (refer to Additional file 1).

### Study setting

The target population for the second BAP clinical trial is Australian adults, male and female, aged 60 years and over, with self-reported history of moderate depressive symptoms. Participants will initially be recruited from the larger BAP cohort using a computer algorithm to identify individuals that meet eligibility criteria. For the second BAP trial, all cohort participants residing in the greater Sydney metropolitan area with an elevated risk of major depression (as evidenced by scores ranging from 16 to 29 on the K-10 at the point of screening for the first BAP trial), will be invited to participate. Given the need to attend for face-to-face clinical assessments it is not feasible to include members of the cohort that reside outside of the Sydney metropolitan area. Of the 24,352 participants in the cohort database, approximately 4,400 showed sub-threshold depressive symptoms previously and approximately 60 % of this subgroup were based in the greater Sydney metropolitan area. Enrichment strategies will be implemented through the Australian Electoral Commission, as per our previous methods, should our recruitment projections be inaccurate. Individuals will be required to provide informed consent prior to study participation.

### Eligibility criteria

Participants eligible for the second BAP clinical trial must comply with all of the following criteria at the point of randomisation:

1. Have provided written informed consent before any trial procedures are undertaken,
2. Be aged 60 years or older, and
3. Have a lifetime history of depressive symptoms (defined as a K-10 score ranging from 16 to 29), meaning that participants included in the trial will be at greater risk of depression.

Participants will be excluded from participating in the second BAP trial if at the point of randomisation they:

1. Have a prior history of stroke or other serious cerebrovascular or cardiovascular disease, neurological disease, significant psychiatric disease (other than major depression) or neurodegenerative disease;
2. Demonstrate clinically significant current depressive symptoms (as defined by a summed PRIME-MD Patient Health Questionnaire (PHQ-9) score of ≥10, which includes a score of ‘2’ or ‘3’ on the 0 to 3 Likert scale on one and/or both of the cardinal depression symptoms on the PHQ-9 (that is, question 1 and/or question 2)) at the time of eligibility screening;
3. Receive a diagnosis of current major depression based on the psychiatric structured diagnostic interview conducted during the baseline medical assessment;
4. Demonstrate suicidal ideation (as defined by a response of either ‘2’ or ‘3’ on the 0-3 Likert scale at question nine of the PHQ-9) at the time of eligibility screening or as determined during the psychiatric structured diagnostic interview conducted during the baseline medical assessment;
5. Are currently taking antidepressant medication or any supplement containing omega-3 fatty acids, or any other medications that are contraindicated with either of the pharmacological treatments under study.

### Interventions

All eligible participants will be randomised on a 1:1:1 basis to either one of the two novel pharmacotherapy treatment arms (each with a placebo counterpart) or to the placebo arm. As such, all participants will take two types of trial treatments over the course of the treatment period (that is, either one active and one placebo, or both placebo variants) as follows:

1. Omega-3 fatty acid intervention, 1,200 mg EPA and 800 mg DHA daily: This dosage combination has been designed to be consistent with our previous work using magnetic resonance imaging (MRI) [55] and is also aligned with a number of other findings in this area [38, 40] as well as the *National Heart Foundation of Australia Guidelines* [76]. This dose and combination of EPA and DHA is also associated with a minimal side-effect profile [77]. Participants in this intervention arm will take four deodorised omega-3 fatty acid capsules each day and one microcrystalline cellulose placebo capsule each morning.
2. Sertraline intervention, 50 mg daily: This is the recommended intervention for treatment of mild to moderate depression [78]. The dose was selected to lessen potential adverse effects as well as for ease of administration [65, 67]. For the purposes of the trial, sertraline tablets will be encapsulated and microcrystalline cellulose will be added to the capsule to prevent the tablet from rattling within. Participants will take one encapsulated sertraline tablet each morning, as well as four paraffin oil placebo capsules each day.
3. Placebo intervention, daily: Participants in the control arm will take four paraffin oil placebo capsules each day and one microcrystalline cellulose placebo capsule each morning. For the purposes of the trial, the paraffin oil capsules will be manufactured to look identical in appearance and weight to their active omega-3 fatty acid counterparts. The paraffin oil capsules will also contain small amount (not more than 5 mg) each of EPA and DHA to ensure a consistent flavour and smell between the active and placebo counterparts. The microcrystalline cellulose placebo capsule will be manufactured to look identical in appearance and weight to the active sertraline counterpart.

All randomised participants will receive the capsules according to the same monthly schedule, irrespective of the intervention arm assigned. They will be provided at face-to-face interview each month for the first three months and then mailed in special packaging to participants’ homes in three monthly instalments subsequently thereafter. Participants will self-administer four oil capsules (either fish oil or paraffin oil) at any time of the day, and one capsule (either sertraline or microcrystalline cellulose) in the morning with or without food. Participants will be given instructions for administration of all study interventions that will include safety information. Participants will also receive a medication-tracking calendar to aid compliance and recording of any side effects.

**Fig. 1:**
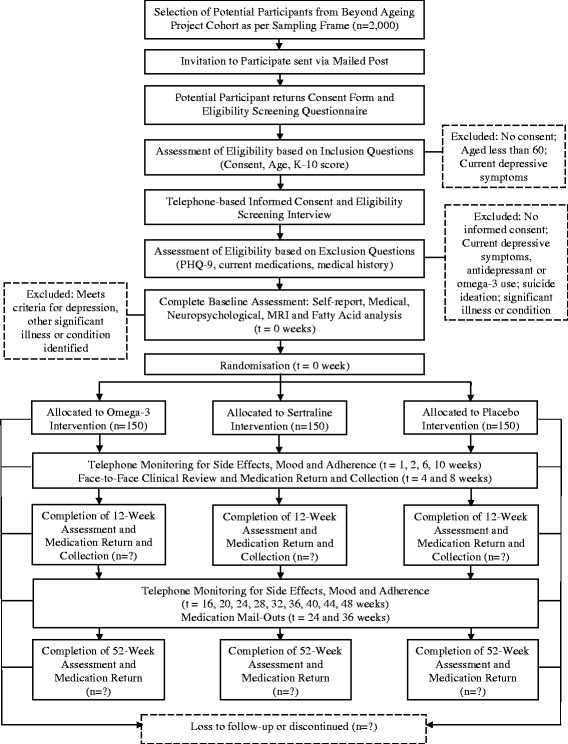
Flow of Participants

Compliance and safety monitoring will be conducted by telephone assessment (as indicated in Fig. 1) and at each scheduled clinical review and follow-up assessment. Compliance will also be assessed by capsule count. Participants will be required to return their medication-tracking calendar, along with any unused capsules at each face-to-face visit where a member of the BAP trial team will record the number of capsules remaining. In addition, all participants will undergo blood testing at baseline, 3 months and 12 months whereupon the amount of fatty acids in the sample will be assessed for the purpose of compliance.

As this is a prevention study, there is no minimum response level. Therefore, the assigned study intervention will not be modified by the trial investigators in any way, unless the participant and/or the BAP Trial Clinician recommend discontinuation on the basis of side effects or harms, or the participant withdraws his/her consent. Where a participant discontinues his/her assigned intervention, all efforts will be made to retain the participant in the trial to enable follow-up data collection and prevent missing data.

During the study, details of current and new concomitant medications will be recorded. A BAP Trial Clinician will be consulted to approve study interventions with all said concomitant medications. Particular attention will be paid to medications likely to interfere with either sertraline or omega-3 fatty acid, as detailed in the Investigators’ Brochure. Participants and their GPs will be informed of all medications contraindicated with sertraline and omega-3 fatty acid supplements upon entry to the trial, and asked to inform the investigators of the commencement of any medication regimes during the study period.

### Outcomes

#### Primary outcome

The primary outcome is the prevention of depressive symptoms over a 12 month period. This will comprise the mean change in severity of depressive symptoms as measured by scores on the PHQ-9 from baseline to 12 months. The PHQ-9 is a nine-item assessment of depressive symptoms, which provides a summary score ranging from zero to 27. The PHQ-9 is a reliable and valid measure of depressive symptoms, which has been widely used in both clinical and epidemiological studies of people with depressive symptoms and is sensitive to change [79, 80]. This self-report measure will be embedded into each of the formal assessments at baseline, three months and 12 months.

#### Secondary outcomes

Secondary outcomes will comprise: a) prevention of depression onset at 12 months; b) reduction in cognitive decline over 12 months; and c) changes in brain neurometabolites and volume over 3 months.

- Prevention of depression onset: The prevention of depression onset will be indicated by the difference in the proportion of participants in each intervention arm classed as being asymptomatic for depression at 12 months. ‘Depression’ will be defined by caseness, as evidenced by a score ≥10 on the PHQ-9.
- Reduction in cognitive decline: This outcome will comprise the mean change in performance between baseline and 12 months across the domains of processing speed (Cambridge Neuropsychological Test Automated Battery (CANTAB) Reaction Time test [81] and the Trailmaking Test Part A [82]) and memory (CANTAB Paired Associate Learning test [81] and the Rey Auditory Verbal Learning Test (RAVLT) [83]).
- Brain neurometabolites and volume: This outcome comprises the change in concentration of key metabolites in the hippocampus (NAA) and anterior cingulate (GSH) from baseline to three months (as measured *in vivo* using magnetic resonance spectroscopy (MRS), as well as changes in the volume of the hippocampus at three months).

#### Other outcomes

Other outcomes measured at baseline, three months and 12 months include:

- Other binary depression outcome measures that can be constructed from the PHQ-9 items including ‘clinically significant magnitude of change’ and the number needed to treat;
- Executive functioning (the Trailmaking Test Part B [82], Delis-Kaplan Executive Function System (DKEFS) Stroop Task [84] and the Controlled Oral Word Association Test (COWAT) [84]);
- Anxiety (using the Generalised Anxiety Disorder Scale, GAD-7 [85]);
- Sleep quality (using the Pittsburgh Sleep Quality Index, PSQI [86]); and
- Disability (using the World Health Organisation Disability Assessment Scale, WHODAS [87]).

In addition, demographic, health and lifestyle factors will be described by treatment group at trial entry and overall at three months and 12 months.

### Participant timeline

Participants identified in accordance with the pre-specified sampling frame (see Table 1) will be contacted by mail and invited to participate in the second BAP trial. The mailed recruitment package will contain a letter of invitation, the Participant Information Statement and Consent Form, an eligibility-screening questionnaire addressing current depressive symptoms and a reply-paid envelope to return the required forms to the Brain & Mind Research Institute. The Participant Information Statement provides a detailed description of the BAP trial, the procedures and time involved in participating, information on the three treatments groups and randomisation process each participant will undergo to either one of these groups, confidentiality information and contact information for the trial team should potential participants want further information. In providing consent, participants will be required to enter their full name and sign and date the form. An independent witness is also required to sign and date the form, confirming the potential participant is acting rationally and voluntarily.

**Table 1:** Sampling framework to identify potential participants in the Beyond Ageing Project (BAP) cohort

Inclusion Criteria	Specification
Age at entry to the BAP cohort	Aged ≥60 years
Self-reported sub-threshold depressive symptoms at entry to the BAP cohort	Where responses to all ten items on the K-10 are summed (where ‘all of the time’ = 5 and ‘none of the time’ = 1) and the summed score ranges from 16 to 29
Resides in Sydney, New South Wales	Has a postcode in the range of 1000-1920; 2000-2249, 2555-2574; or 2740-2786
Does not meet criteria for any of the following *Exclusion Criteria*:	
• Self-reported history of serious cerebrovascular or cardiovascular illness at entry to the BAP cohort	Doctor’s diagnosis of any of the following problems that are not presently under control:
	• Serious heart problems
	• Diabetes
	• High blood pressure
	• Severe blockage of a lung artery
	Severe chest pains that are brought on by physical activity or occur while at rest
	Doctor’s diagnosis of stroke
	Doctor’s diagnosis of mini-stroke or Transient Ischaemic Attack
	Heart problem in the last 12 months that led to a hospital admission, hospital emergency contact or consultation with a specialist
• Self-reported neurological illness at entry to the BAP cohort	Ever had an epileptic fit
	Currently have epilepsy
	Ever had bleeding in the eye
	Ever had a serious head injury that interfered with memory, resulted in loss of consciousness or caused a blood clot in the brain
• Self-reported psychiatric illness at entry to the BAP cohort	Doctor’s diagnosis of bipolar disorder or mania
	Summed K-10 score ranging from 30 to 50 (suggesting clinical management of depression presently required, rather than preventative management)
• Self-reported neurodegenerative disease at entry to the BAP cohort	Doctor’s diagnosis of dementia or Alzheimer’s disease
• Other self-reported significant illness at entry to the BAP cohort	Doctor’s diagnosis of brain tumour in the last 12 months
	Currently have:
	• Cancer
	• Alcohol and/or drug abuse
	• Kidney disease
	• Liver disease
	• Anaemia or other blood disease

Upon receipt of completed consent forms and eligibility screening questionnaires, a member of the BAP trial team will assess the potential participant’s eligibility against the trial inclusion criteria. Those who do not meet criteria will receive a letter outlining why they have not been selected to participate. Any potential participant who is identified in the eligibility-screening questionnaire as being at risk of self-harm or currently exhibiting clinically significant depressive symptoms will be contacted by a BAP Trial Clinician for referral to clinical services. Those who meet trial inclusion criteria will be contacted via telephone where further information about the BAP trial will be provided and informed consent will be undertaken. Potential participants will undergo further eligibility screening, whereby the potential participant’s eligibility will be assessed against the trial exclusion criteria. Participants who do not meet criteria, due to a prior history of stroke or other serious cerebrovascular or cardiovascular disease, neurological disease, significant psychiatric disease (other than depression) or neurodegenerative disease, or due to current antidepressant use or omega-3 fatty acid supplementation, will be thanked for their time and will not participate further. Any participant who is already taking an omega-3 fatty acid supplement but indicates a willingness to cease the supplement for the duration of the BAP trial will be instructed to undergo a washout period of at least eight weeks prior to baseline assessment and randomisation.

Following telephone screening, all eligible participants will be invited to attend the Trial Coordinating Centre (Brain & Mind Research Institute) for baseline assessment. One week prior to attendance, participants will be mailed a self-report questionnaire, which they will complete in their own time. The baseline assessment will comprise the following: self-report questionnaire, medical assessment, neuropsychological assessment, blood sampling and MRS. Ongoing participation in the trial will be measured from this time-point.

### Sample size

We will randomly select 2,000 of those meeting trial eligibility criteria from the broader BAP cohort to participate. Even though this cohort has expressed a willingness to participate in research, we expect a conservative response rate of 50 % from this initial invitation. Assuming 65 % of those who provide consent will be eligible to participate, this leaves a potential sample of 650. Taking into account a 10 % attrition rate post-randomisation (in accordance with the first BAP prevention trial), this will provide a final sample of approximately 585.

We will attempt to recruit a minimum of 450 participants with 150 per group (135 per group with 10 % attrition) for the comparison of endpoint depression scores between the omega-3 fatty acid and sertraline interventions and the placebo condition. Assuming a correlation of 0.7 between baseline and endpoint depression measures and constancy of dispersion, the standard deviation of difference scores is 0.77 × the standard deviation of the raw scores. In a two-tailed test of contrasting differences between the two treatment arms, not relying on pooling error sums with the third treatment arms with alpha = 0.05 and power 80 %, the forecast number of participants can detect differences in depression change scores of 0.34 standard deviations. This equates to approximately 0.25 standard deviations difference in raw depression scores.

### Recruitment

Trial participants will be recruited into the study in a series of waves over 48 months, a procedure utilised in the first BAP prevention trial. The entire study is expected to be complete within five years.

### Allocation

The allocation of participants to an intervention arm will be achieved through a centrally coordinated randomisation service provided by the National Health and Medical Research Council (NHMRC) Clinical Trials Centre, which operates independently of the BAP Trial Coordinating Centre and all trial personnel in day-to-day contact with participants. Once informed consent is obtained and a BAP Trial Clinician has assessed eligibility and made the decision to enrol a participant, a BAP Research Officer will telephone the Interactive Voice Response System (IVRS) to perform the random allocation. Confirmation of each allocation procedure will be automatically emailed to the BAP Trial Coordinating Centre.

### Sequence generation

The randomisation procedure will be based on variable length permuted blocks. Eligible participants will be stratified according to sex and presenting depression severity (their summed K-10 score on the baseline self-report questionnaire). The last factor is used to ensure that the ‘tail’ of participants with higher levels of symptoms is distributed equally across the three conditions. Symptom severity will be classified in accordance with the 2007 National Health Survey [88], where K-10 total scores were collapsed into four categories including (*low*, scores 10-15; *moderate*, scores 16-21; *high*, scores 22-29; and *very high*, scores 30-50). For the purposes of stratification, scores ranging from 10-15 will be classified as low, and scores ranging from 16 to 50 as high. The allocation sequences within each strata will be generated by a NHMRC Clinical Trials Centre Biostatistician and loaded into the IVRS prior to trial commencement.

### Allocation concealment mechanism

The allocation codes will be documented by the NHMRC Clinical Trials Centre, not to be provided until the completion of the trial, unless unblinding is required in an emergency. The allocation code assigned to any participant will only be broken by the Chief Investigator or authorised person if the patient’s safety is at risk and it is absolutely necessary to ascertain the type of treatment given. The IVRS incorporates an unblinding service available 24 h a day that can only be accessed by authorised persons. Where patient safety is under consideration, ceasing the trial treatments will be the preferred management option in contrast to unblinding.

### Blinding (masking)

This is a double-blind clinical trial whereby the study participants and the clinical investigators/trial monitoring staff will be unaware of the treatment assignment. Only the BAP Trial Statistician will have access to unblinded data at the individual level. However, he will not have any direct contact with trial participants. Several strategies will be employed to minimise bias through accidental unblinding.

As the two pharmacological treatments under investigation are different in appearance, blinding will be ensured by a double-dummy design. This will involve giving all participants two formulations: one group will receive the active omega-3 fatty acid treatment plus a placebo of the sertraline treatment; a second group will receive the opposite combination (active sertraline plus a placebo of the omega-3 fatty acid treatment), while the third group will receive a placebo version of both omega-3 fatty acid and sertraline.

Additionally, in order to protect the blinding of both participants and investigators, the active and placebo variants of each treatment will appear identical in appearance and weight to their active counterparts. As previously described, the paraffin oil capsules will also contain small amount each of EPA and DHA to ensure a consistent flavour and smell between the omega-3 fatty acid and placebo counterparts. The packaging and labelling of each active and placebo intervention will also be identical in weight, size and appearance.

Clinical outcomes will be assessed by personnel who are unaware of the participant’s treatment allocation. The Trial Coordinator will maintain an unblinding log to record any specific personnel that may be accidentally (or deliberately) unblinded during the course of the trial, along with the date, scope and reason for doing so.

The routine collection of blood for the purpose of safety monitoring and adherence to treatment will be analysed by a central independent laboratory. In order to preserve the blinded status of clinical investigators, summary reports indicating whether a participants results fall within a pre-specified range will be provided for the purposes of monitoring safety. Samples collected for the purpose of adherence monitoring will not be analysed until the completion of the trial.

### Data collection methods

Table 2 provides an overview of the timeframe for assessment and the measures that are used. All self-report measures will be collected via hardcopy questionnaires that will be mailed to participants for completion in their homes at baseline, three month and 12 month time-points. Participants will return the completed questionnaires to the Trial Coordinating Centre.

**Table 2:** Complete list of outcome measures and assessments

	Recruitment	Screening/baseline assessment	Visit 1	Visit 2	Monitoring calls	Three month assessment	12 month assessment	Early termination
Week(s):	−6 to -2	−2 to 0	4	8	1, 2, 6, 10 then monthly	12	52	
Informed consent	X							
Inclusion/exclusion criteria		X						
Self-report (includes PHQ-9, K-10, GAD-7, WHODAS, PSQI)		X				X	X	
Medical assessment (includes semi-structured psychiatric interview)		X				X	X	X
Neuropsychological assessment (includes CANTAB RT and PAL, Trailmaking A & B, RAVLT, DKEFS, COWAT)		X				X	X	
MRS assessment		X				X	X	
Clinical review and medication collection and return		X^a^	X^a^	X^a^		X^b^		
Compliance (capsule count)			X	X	X	X	X	
Compliance (fatty acid blood analysis)		X				X	X	
Monitor for depressive symptoms and suicidality		X	X	X	X	X	X	X
Monitor adverse events			X	X	X	X	X	X
Mail out of medications	Months seven and 10 (three month supply)							
Early termination/trial cessation follow-up phone call	Within three to five days following treatment cessation							

*PHQ-9* Patient Health Questionnaire, *K-10* Kessler-10 Psychological Distress Scale, *GAD-7* Generalised Anxiety Disorder Scale, *WHODAS* World Health Organisation Disability Assessment Scale, *PSQI* Pittsburgh Sleep Quality Index, *CANTAB RT and PAL* Cambridge Neuropsychological Test Automated Battery Reaction Time Test and Paired Associate Learning Test, *RAVLT* Rey Auditory Verbal Learning Test, *DKEFS* Delis-Kaplan Executive Function System Stroop Task, *COWAT* Controlled Oral Word Association Test, *MRS* Magnetic resonance spectroscopy

^a^ One month supply

^b^ Three month supply

All clinical (medical, psychiatric and neuropsychological) measures will be collected using standardised instruments by staff trained in the trial protocol and procedures. All clinical assessments, including MRS and MRI, will be performed at the Trial Coordinating Centre. Clinicians will be given a hardcopy case record form (CRF) in which they will document results. Clinicians will be required to sign-off on the CRF on completion of the assessment.

All MRS data will be transferred offline for post-processing immediately following acquisition. Metabolite concentrations will be determined using the LCModel software package [89].

Drug dispensing and returns will be documented on individual hardcopy investigational product accountability forms. Reasons for exclusion, withdrawal or loss to follow-up will also be recorded.

### Data management

All data will be entered electronically into the master trial database by suitably qualified staff, supervised by the Trial Coordinator. Data will be entered into a user-friendly Filemaker platform, and integrity will be enforced through a variety of mechanisms, including referential data rules, valid values, range checks and consistency checks. The option to choose a value from a list of valid codes will also be available when applicable. The master trial database will be held on a secure server hosted by the Faculty of Medicine, University of Sydney. The trial database will be password protected, and only authorised trial personnel will have access. The database will be maintained in accordance with University of Sydney ICT policy and procedures. Upon database lock, data will be exported to a statistical software package for subsequent analysis.

### Statistical methods

Primary outcome analysis will be undertaken on an intention-to-treat basis. Mixed-model repeated measures analyses will be used because of the ability of this approach to include participants with missing data [90]. Characteristics of the participants including sociodemographics, disability, physical activity, sleep and health and lifestyle factors will also be described by treatment group and overall at three and 12 months. Safety criteria including adverse events, laboratory parameters and withdrawals will also be analysed. The type one error will be set at 5 %. Descriptive statistics will include number of participants (N), mean, standard deviation, minimum and maximum and, if a non-normal distribution, the median and first/third quartiles will be reported.

### Data monitoring

The accuracy, completeness and progress of data will be monitored continuously throughout the trial. Each CRF will have a data checklist attached, on which the assessments completed, drugs dispensed and other information will be recorded. Any missing information or illegible data will be queried with the researcher, clinician or interviewer responsible for its collection. Audits will be undertaken periodically to ensure that all data relating to informed consent, self-report questionnaires, medical and neuropsychological assessment, MRS, blood tests and dispensing, are accounted for. All original CRFs will be stored securely by trial identification number at the Trial Coordinating Centre. Following data entry into the master trial database, data will be randomly checked for accuracy and completeness and verified against source data. Approximately 5 % of participants will be specifically monitored.

### Safety and event monitoring

An independent Data and Safety Monitoring Committee will be established to safeguard the patients’ interests and to evaluate formally on a regular basis whether, for any reason, they would recommend that the study be modified or stopped. Two formal interim analyses are planned in accordance with the Data and Safety Monitoring Committee Charter. The Trial Management Committee, the collaborators, the study sponsor and all the project staff, with the exception of the unblinded statistician, will remain ignorant of the interim results.

### Harms

During the course of the study, participants will be assessed for adverse events (AEs) using pre-specified checklists comprising both expected (as per the Investigators’ Brochure) and unexpected adverse events. Clinician-rated assessments will be undertaken at the one and two month brief review, as well as at the three and 12 month follow-up assessment. In addition, participant self-reports will be completed at each of the same time-points. In accordance with evidence linking the use of some selective serotonin reuptake inhibitors such as sertraline, with an increased risk of hyponatraemia (low sodium concentration in blood) in elderly populations [91] particularly in the first few weeks of treatment, all study subjects will undergo electrolyte testing in the weeks following randomisation. In addition to these clinical and laboratory measurements, research assistants conducting safety monitoring and adherence telephone interviews at one, two, six, and ten weeks and then monthly to 12 months, will also assess for the occurrence of adverse events using the participant self-report checklist.

All AEs (including serious adverse events, or SAEs), regardless of relatedness to the investigational products, will be recorded on an adverse event form. Recording will be completed in a concise manner using standard, acceptable medical terms. Surgical interventions, which are planned prior to study enrolment, will not be considered as AEs. All AEs will be classified and graded in accordance with the Common Terminology Criteria for Adverse Events (Cancer Therapy Evaluation Program, Version 3.0, 2006).

### Study sponsorship and organisation

The sponsor of the trial is the University of Sydney. For the purposes of the trial, EPAX 6000 TG/N (raw omega-3 fatty acid) will be supplied in-kind by Pathway International Pty Ltd and manufactured by LIPA Pharmaceuticals. LIPA Pharmaceuticals will also manufacture the matching paraffin oil capsules. Setrona™ (sertraline hydrochloride) tablets will be purchased from the Royal Adelaide Hospital Department of Pharmacy and encapsulated by Pharmaceutical Packaging Professionals Pty Ltd. Pharmaceutical Packaging Professionals will also prepare the matching microcrystalline cellulose placebo capsules, and provide packaging and labelling for all of the investigational products. The trial is supported by the Bupa Health Foundation’s Healthy Ageing initiative, along with the National Health and Medical Research Council of Australia, through a project grant awarded to the Brain & Mind Research Institute. The trial is being coordinated independently of the funding bodies and suppliers of the investigational products, by the Brain & Mind Research Institute, Sydney, Australia and overseen by the Trial Management Committee.

### Auditing

The Trial Management Committee and investigator team will allow independent monitors (for example, regulatory agencies) to visit the site and facilities where the trial will take place in order to ensure compliance with the protocol requirements at regular intervals. The sponsor may also conduct monitoring at regular intervals, depending on recruitment the rate, and as arranged between the investigator team and the sponsor.

### Ethical considerations

The trial will be undertaken in compliance with the World Medical Association Declaration of Helsinki (revised version of Seoul, 2008), international standards of Good Clinical Practice and the applicable regulatory requirements in Australia. The design and implementation of the trial has been approved by The University of Sydney Human Research Ethics Committee (Reference Number: 12-2009/12097). All important protocol modifications will be communicated to the Human Research Ethics Committee prior to implementation.

## Discussion

LLD is associated with significant morbidity and mortality, and contributes to cognitive decline [4] and disability [6] that is unlikely to be reversible. Even with optimal treatment of LLD, only 34 % of the disease burden in the form of ‘years lived with disability’ could be averted [7]. The current health, social and economic costs of depression make prevention imperative from a public health perspective [92]. While effective treatments for depression are available, as many as 50 % of patients will not achieve remission with their first treatment [93] and ultimately 20 to 30 % will not achieve full recovery [94]. Those who do achieve full recovery remain at high risk for further episodes [1]. Furthermore, epidemiological data has shown that if depressive symptoms in older people were averted, the prevalence of dementia could be reduced by up to 10 % [16]. Therefore, prevention of this disease is of utmost importance.

This innovative trial aims to address the long-neglected area of prevention of depression in older adults, and represents an opportunity to test two novel pharmacological approaches that are particularly relevant and acceptable to this ‘at risk’ cohort. The interventions are targeted to the pathophysiology of LLD, and regardless of the effect size of the intervention, the outcomes of this study will offer major scientific advances regarding the neurobiological action of these agents. Importantly, omega-3 fatty acids are a low cost and low risk prevention dietary supplement that are already widely used in the community, but have not been evaluated for their prevention capacity. As sertraline is the most widely prescribed antidepressant in general practice, findings from this trial may be directly translatable to clinical practice. Benefits are expected for depressive symptoms and cognition, and also for broader psychosocial functioning. If effective, this intervention could feasibly be delivered on a larger scale with the potential to result in significant reductions in the high healthcare utilisation and costs associated with depression.

## Trial status

Recruitment to the second BAP trial commenced in July 2011. Recruitment is currently ongoing, and the last participant randomised is expected to complete his/her 12-month follow-up in late 2016.

## Additional file

Additional file 1:
**Flow of participants.** Graphical representation of the flow of participants through the Beyond Ageing Project Phase 2.

## Abbreviations

ACTRN: Australian and New Zealand clinical trial registry number
AE: Adverse event
BAP: Beyond Ageing Project
BDNF: Brain-derived neurotrophic factor
CANTAB: Cambridge neuropsychological test automated battery
CBT: Cognitive behavioural therapy
COWAT: Controlled oral word association test
CRF: Case record form
DKEFS: Delis-Kaplan executive function system
DHA: Docosahexaenoic acid
DSM-IV: Diagnostic and Statistical Manual of Mental Disorders, 4^th^ edition
EPA: Eicosapentaenoic acid
GAD-7: Generalised anxiety disorder scale
GSH: Glutathione
HPA: Hypothalamus-pituitary-adrenal
IVRS: Interactive voice response system
K-10: Kessler-10 questionnaire
LLD: Late-life depression
MD: Major depression
MRI: Magnetic resonance imaging
MRS: Magnetic resonance spectroscopy
NAA: Nacetylaspartate
NHMRC: National Health and Medical Research Council
PHQ-9: Patient health questionnaire
PSQI: Pittsburgh sleep quality index
RAVLT: Rey auditory verbal learning test
RCT: Randomised controlled trial
SAE: Serious adverse event
SSRI: Selective serotonin re-uptake inhibitor
WHODAS: World Health Organisation disability assessment scale
